# Use of multiple methods for genotyping *Fusarium *during an outbreak of contact lens associated fungal keratitis in Singapore

**DOI:** 10.1186/1471-2334-8-92

**Published:** 2008-07-15

**Authors:** Roland Jureen, Tse H Koh, Grace Wang, Louis YA Chai, Ai L Tan, Tracy Chai, Yong W Wong, Yue Wang, Paul A Tambyah, Roger Beuerman, Donald Tan

**Affiliations:** 1 Department of Laboratory Medicine, Alexandra Hospital, Singapore; 2 Department of Pathology, Singapore General Hospital, Singapore; 3 Department of Medicine, National University Hospital, Singapore; 4 Singapore Eye Research Institute, Singapore; 5 Candida albicans Molecular & Cell Biology Laboratory, Institute of Molecular and Cell Biology, Singapore

## Abstract

**Background:**

In Singapore, an outbreak of fungal keratitis caused by members of the *Fusarium solani *species complex (FSSC) was identified in March 2005 to May 2006 involving 66 patients. Epidemiological investigations have indicated that improper contact lens wear and the use of specific contact lens solutions were risk factors.

**Methods:**

We assessed the genetic diversity of the isolates using AFLP, Rep-PCR, and ERIC-PCR and compared the usefulness of these typing schemes to characterize the isolates.

**Results:**

AFLP was the most discriminative typing scheme and appears to group FSSC from eye infections and from other infections differently.

**Conclusion:**

There was a high genomic heterogeneity among the isolates confirming that this was not a point source outbreak.

## Background

*Fusarium *spp. are filamentous fungi commonly isolated from environmental sources such as soil, plant roots, plant debris and water systems [1,2]. They may cause invasive infections predominantly in immunocompromised persons [2]. *Fusarium *species can also cause ocular infections, usually keratitis or endophtalmitis, in immunocompetent persons usually associated with trauma [3-6]. Several morphologically similar species are grouped together in the *Fusariun solani *species complex (FSSC), but further genotypic characterization of species within this complex is laborious and is usually not done routinely in clinical laboratories. Members of this species complex are usually reported in the literature as *Fusarim solani *[7]. Nucleic acid based methods are often used in laboratories to identify *Fusarium *spp. [8-10].

Several different methods have been used for molecular typing of fungal isolates associated with outbreaks. Godoy et. al. showed that an enterobacterial repetitive intergenic consensus PCR (ERIC-PCR) and PCR restriction fragment length polymorphism (PCR-RFLP) were useful for genotyping *Fusarium *isolates [5]. Other methods that have been used include REP-PCR [11], amplified fragment length polymorphism (AFLP) [12,13], and multilocus sequence typing (MLST) [3,14]. Recently, microsphere array-based genotyping was also described [7].

In Singapore, there was an outbreak of fungal keratitis caused by members of the FSSC from March 2005 to May 2006 involving 66 patients. Epidemiological investigations in Singapore and the U.S. indicated that improper contact lens wear and the use of ReNu contact lens solution were risk factors and these findings have previously been published [3,4,15]. At the same time a similar outbreak of *Fusarium *keratitis associated with contact lens use was also described in Hong Kong [3]. An extensive genotyping study including the *Fusarium *isolates involved in the U.S. outbreak was recently reported [7].

The present study describes molecular genotyping of isolates from the Singapore outbreak and also includes some epidemiologically unrelated isolates from patients with non-eye associated infections for comparative purposes. The aims of the study were: (1) To elucidate the genetic diversity of the isolates, and (2) to compare the discriminatory power and concordance of strain typing systems using AFLP, Rep-PCR, and ERIC-PCR.

## Methods

### Isolation and identification

Clinical samples obtained from patients and their contact lenses or lens cases were cultured on blood agar, chocolate agar, Sabouraud dextrose agar, and thioglycollate broth. Suspected *Fusarium *isolates were identified to species complex using 28S rRNA gene sequencing as previously described [9]. Written or verbal consent was obtained from all patients or the parents of patients younger then 21 years. Institutional Review Board approval was provided by the Institutional Review Board of the Singapore National Eye Center.

### *Fusarium *isolates

A total of 65 isolates were included in the study. Sixty-two were members of the FSSC, in addition there was one each of *F. cf. incarnatum*, *F. oxysporum *and one isolate that initially had been misclassified and finally was identified as *Melanospora fallax*. None of the three non-FSSC isolates were outbreak isolates. Only one isolate per patient was included in the study. Thirty-six originated from cornea or cornea scraping, three were from eye without further specification, seven were from contact lenses, five were from contact lens cases, one was from contact lens case solution, eight were from nails, three were from skin lesions, one was from a wound, and one was from blood. Of the 65 isolates, 61 were from Singapore, three were kindly sent to us by colleagues in Malaysia, and one was the FSSC ATCC 36031 strain originally isolated from a corneal ulcer in a patient from Nigeria. The isolates are housed at Department of Pathology, Singapore General Hospital and will be made available to researchers upon request.

### DNA extraction

DNA was extracted from the mycelium using the Ultra Clean soil DNA isolation kit (MoBio Inc., Solana Beach, CA). The protocol recommended by the manufacturer was followed, using the alternative protocol for maximum yields. This method has previously worked well with *A. fumigatus *in a comparison of six DNA extraction methods performed by Fredricks et al. [16].

### Amplified fragment gel electrophoresis (AFLP)

AFLP was performed using the AFLP™ Microbial Fingerprinting kit from Applied Biosystems (Applied Biosystems, Foster City, CA). Mse1 and EcoR1 enzymes were used for restriction. Preselective amplification was conducted with EcoR1+0, and Mse1+0 primers. Selective amplification was then performed using EcoR1+AA, and Mse1+CA primers as outlined in the kit manual. For the amplification steps all isolates were run in the same thermocycler block (model 9600, Applied Biosystems). The amplification products were run on a DNA sequencer (ABI Prism 310, Applied Biosystems). For this 1 μl of reaction mixture was mixed with 12 μl deionized formamide and 0.25 μl GeneScan-500 (ROX) size standard (Applied Biosystems). GeneScan collection software (Applied Biosystems) was used to collect data. After tracking and extraction of lanes, data were exported to the BioNumerics software version 4.6 (Applied Maths, Sint-Martens-Latem, Belgium) for further analysis. Normalisation was done by use of the reference positions of the internal DNA marker GeneScan-500 (ROX). Fragments ranging in size from 50 to 500 bp were used for comparison. The Pearson coefficient of similarity of AFLP curves was calculated, and UPGMA was used for cluster analysis. When five isolates were run in triplicate a similarity value of ≥ 95% was obtained. Thus two isolates were considered to have identical AFLP patterns if the similarity was ≥ 95%.

To minimize the possibility of inclusion of an isolate to a group because of test variability and in line with previous investigations on bacteria [17] and *Fusarium *[13], isolates were assigned to the same group if the AFLP pattern was at least 90% similar.

### Rep-PCR

Rep-PCR was performed as previously described [11] with minor modifications. In brief, PCR reactions were performed in a total volume of 25 μl. The PCR mixture consisted of 2.5 μl of 10× PCR buffer, 2 μl of Rep-PCR primer: 5'-RCGYCTTATCMGGCCTAC-3', 0.5 μl of dNTP mixture (10 mM), 0.125 μl of Taq polymerase (Qiagen GmbH, Hilden, Germany) and 2 μl of genomic DNA. Final Mg^2+ ^concentration was 2 mM. PCR was performed as follows: 1 cycle of 94°C for 2 min, then 35 cycles of 94°C for 30 sec, 40°C for 30 sec, 70°C for 1 min 30 sec, followed by a 3 min extension time at 70°C (Biometra T3 thermocycler, Göttingen, Germany). The amplified products were analyzed by electrophoresis in a 1.2% agarose gel using ethidium bromide staining. Analysis of gel patterns was performed by visualization of band patterns on gels from each isolate, which were then compared for relatedness to all other isolates. Isolates with identical patterns were assigned to the same REP-PCR group.

### ERIC-PCR

ERIC-PCR was performed as previously described [5], using ERIC-1 and ERIC-2 primers described by Versalovic et al. [11]. Fingerprints were assigned to a different type if any band differences were observed.

### Comparison of methods

Discriminatory power, i.e. the ability of a typing system to discriminate between unrelated strains [18,19], was measured using Simpson's index of diversity [20]. Simpson's mathematical formulas enable us to calculate the probability that two unrelated strains sampled from the test population will be placed into different groups. This index of diversity has been applied to compare typing methods in order to select the most discriminatory system [19]. A Simpson's index close to zero indicates that there is little diversity as shown by the typing method (index = 0 indicates no diversity at all) whereas a Simpson's index approaching 1 indicates a high diversity as shown by the typing technique (index = 1 indicates maximum diversity where no two isolates are similar). An approximate 95% confidence interval was calculated as proposed by Grundmann et al. [21].

Typing system concordance refers to the concordance of the results by independent typing systems [22]. For this we compared how many isolates that grouped together in one method were grouped in different groups with the other methods. Groups containing two or more isolates were included in the comparision.

## Results

### AFLP

**Figure 1:**
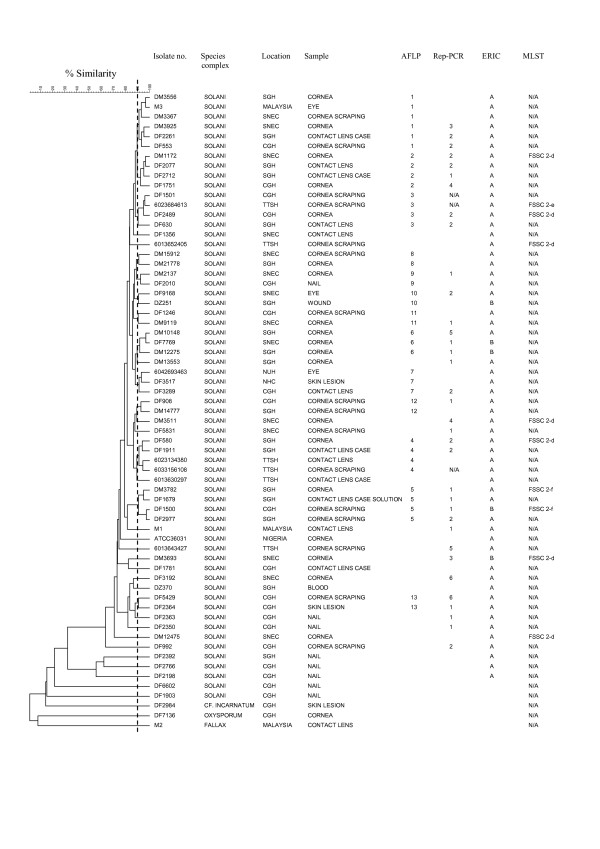
**AFLP dendrogram of 65 isolates produced following Pearson and UPGMA analysis.** Thirteen AFLP groups comprising at least two isolates were formed at > 90% similarity. Distribution of the isolates according to species complex, location, sample, AFLP group, Rep-PCR group, ERIC-PCR group (only groups with two or more isolates are given). MLST results are from reference 3. N/A = no data. CGH = Changi General Hospital, NHC = National Heart Centre, NUH = National University Hospital, SGH = Singapore General Hospital, SNEC = Singapore National Eye Centre, TTSH = Tan Tock Seng Hospital. Fallax refers to *Melanospora fallax*.

The 65 isolates comprised 56 different profiles with less than 95% similarity. When examining isolates sharing at least 90% of the restriction fragments, 38 groups were discerned. Thirteen of these groups contained two or more isolates with six isolates being the maximum in one group (Fig. 1.). Among these thirteen groups there were in total 40 isolates of which 36 were from eye-related specimens (eye, cornea, cornea scraping, contact lens, contact lens case, contact lens case solution). Among the 25 isolates that were not included in these thirteen groups were the *F. cf. incarnatum*, *F. oxysporum*, and *M. fallax *isolates as well as the FSSC ATCC strain. Also not included were nine non-eye associated isolates (nail, skin, wound, blood). The difference in grouping of eye and non-eye related isolates was statistically significant (Chi-square test 6.5, p < 0.05).

### Rep-PCR

The sixty-two isolates typed comprised 33 groups. Six of these groups contained two or more isolates with 15 isolates being the maximum in one group (Fig 1.). Among these six groups there were in total 35 isolates, of which 32 were from eye related specimens (defined as above). Among the 27 isolates that were not included in these six groups were the *F. cf. incarnatum*, *F. oxysporum*, and *M. fallax *isolates as well as the FSSC ATCC strain. Also not included were ten non-eye associated isolates (nail, skin, wound, blood). The difference in grouping of eye and non-eye related isolates was statistically significant (Chi-square test 7.45, p < 0.01).

### ERIC-PCR

The 65 isolates typed comprised seven different groups. *F. cf. incarnatum*, *F. oxysporum*, and *M. fallax *isolates were distinguishable and all FSSC isolates fell into four groups. Two of these groups contained more than one isolate, with 55 isolates in Group A and five in Group B (Fig 1.). The FSSC ATCC strain was included into group A.

### Comparison of methods

Simpson's index of diversity, with confidence intervals was 0.98 ± 0.01 for AFLP, 0.91± 0.05 for Rep-PCR, and 0.28 ± 0.14 for ERIC-PCR. The isolates grouped differently with each method and the concordance of strain groupings based on typing method was low, with 65% and 10% of isolates with identical AFLP type having different Rep-PCR types and ERIC types respectively. Of the isolates with identical Rep-PCR type 63% and 6% had different AFLP and ERIC types respectively. Of isolates with an identical ERIC type, 40% had different AFLP types and 40% had different Rep-PCR types. There appeared to be a statistically significant difference between the grouping of eye related and non-eye related isolates with AFLP and Rep-PCR, but not with ERIC-PCR.

## Discussion

In this study the genetic relationship of *Fusarium *involved in an outbreak of keratitis and other clinical infections were analysed with AFLP, Rep-PCR and ERIC-PCR.

AFLP was the most discriminatory typing scheme of the three we tested. We tried three additional primer sets on a subset of strains in the AFLP amplification step. For one of these primer pairs the discriminatory power was equal to what is presented here, but the other two produced lower discriminatory power (data not shown). This underscores the importance of choice of restriction enzyme, primer and adapter configurations in AFLP to achieve a high discriminatory power [23]. The Rep-PCR scheme we tested was almost as discriminatory as AFLP giving an index of diversity of 0.91 ± 0.05 as compared with 0.98 ± 0.01 for AFLP. Since Rep-PCR is much easier to perform and cheaper, this difference may be perceived as acceptable in a clinical setting when confronted with a suspected *Fusarium *outbreak, especially in smaller clinical laboratories that may not have access to DNA sequencing facilities. However, with Rep-PCR three non eye-related isolates grouped among the eye isolates in the largest group, whereas no non-eye related isolates were included in the five largest AFLP groups. Thus even though Rep-PCR is almost as discriminatory as AFLP, because the eye and non-eye related isolates were distributed differently, it may not be as epidemiologically relevant as AFLP.

The ERIC-PCR in our hands had low discriminatory power (0.28 ± 0.14), and generated only seven different banding patterns. This is in sharp contrast with the study from Brazil by Godoy et al [5]. In their study, using the same protocol, they found 39 groups among the 44 FSSC keratitis isolates. Their isolates were epidemiologically unrelated and came from all over Brazil. In our limited geographical area it is possible that the genetic diversity is at a lower level even though we found substantial diversity using AFLP and Rep-PCR. It is also possible that a limited number of more virulent clones of *Fusarium *is circulating in the local environment that are sufficiently related genetically to be grouped together by AFLP and Rep-PCR, but too similar to be differentiated by the ERIC-PCR. However the ATCC strain, which originally was isolated in Nigeria, is unlikely to be clonally related to the Singapore strains, and ERIC-PCR failed to distinguish this strain from the local strains. ERIC-PCR was also not able to differentiate between eye and non-eye isolates. We therefore conclude that the ERIC-PCR in our hands and in this setting clearly was not discriminatory compared with the AFLP and Rep-PCR schemes.

Concordance between the techniques was low. The fact that both AFLP and Rep-PCR were able to assign eye related and non-eye related isolates differently in our setting could indicate that both techniques may be able to group more dissimilar isolates into meaningful groups, even when the isolates are not very closely related.

Epidemiological investigations into the *Fusarium *outbreak in the United States in 2006 showed that a common source outbreak, or transmission between patients, was unlikely but rather that diverse environmental sources were implicated [3,7]. The epidemiological study of the Singapore outbreak also indicates that the likely mode of acquiring these infections were from environmental sources contaminating a contact lens care solution [4]. The high genetic diversity of the strains in the present study is thus in line with these investigations. Others have reported that this outbreak is most likely due to failure of stressed multipurpose contact lens solutions to eliminate fungal growth [24].

Ten of the corneal isolates included in our study had also been subjected to multilocus sequence typing (MLST) as part of previous investigations in U.S., which found that they all were nested within FSSC group 2 (2-d, 2-e and 2-f) [3]. These 10 isolates were divided into eight groups by AFLP, and five groups by Rep-PCR but Rep-PCR results were only available for nine of the ten isolates (Figure 1). It would from these findings appear that in the local setting AFLP and Rep-PCR may be more discriminative than MLST.

O'Donnell et al. reported in their study, including isolates from the U.S. outbreak, that corneal infections were most frequently associated with FSSC group 1 [7], whereas Zhang et al. in an earlier study reported an association of non-outbreak strains of *Fusarium *with group 3 [14]. The keratitis outbreaks in Singapore, Hong Kong and the U.S. were not associated with trauma but with contact lens use which may explain this discrepant finding. Chang et al. hypothesized that FSSC group 3 isolates may be most commonly associated with ocular trauma [3] and O'Donnell et al. hypothesized that the patients water system may have represented the primary reservoir of infection in the U.S. outbreak [7]. In the present study AFLP typing indicated that eye-related and skin- or nail-related strains were genetically distinct. This could be explained by different FSSC associated with different environments as hypothesized above and indicates that AFLP distinguishes different members of the FSSC.

Many researchers would prefer MLST for epidemiological investigations of FSSC since it provides DNA sequence data [12] and since MLST is more robust when making inter-laboratory comparisons. Our results show however that AFLP was very discriminatory in our setting.

## Conclusion

We conclude that AFLP had the highest discriminatory power of the typing schemes we investigated. There was a high degree of diversity among the *Fusarium *strains included in our study, consistent with earlier reports that this international outbreak was associated with improper contact lens use and a multi-purpose contact lens solution.

## Competing interests

The authors declare that they have no competing interests.

## Authors' contributions

RJ, THK, ALT, YWW, YW, PAT, RB and DT conceived of and designed the study. PAT, RB and DT were the study clinicians. Cultures were analyzed by THK and ALT, the genotyping experiments were conducted by GW, LYAC, TC, and data was analysed by RJ with contributions from THK, PAT and other authors. All authors contributed to the interpretation of the manuscript.

## Pre-publication history

The pre-publication history for this paper can be accessed here:


